# 1-(2-Chloro­phen­yl)-3-(2-ethyl­hexa­noyl)thio­urea

**DOI:** 10.1107/S160053681301828X

**Published:** 2013-07-10

**Authors:** R. Santhakumari, S. Selvanayagam, K. Ramamurthi, N. Radha, Bohari M. Yamin

**Affiliations:** a Department of Physics, Government Arts College for Women (Autonomous), Pudukkottai 622 001, India; b Department of Physics, Kalasalingam University, Krishnankoil 626 126, India; c Department of Physics and Nanotechnology, SRM University, Kattankulathur 603 203, India; d Department of Chemistry, Government Arts College, Karaikudi 630 303, India; e School of Chemical Sciences and Food Technology, Kebangsaan Universiti, Bangi, Selangor 43650, Malaysia

## Abstract

In the title compound, C_15_H_21_ClN_2_OS, the central chromophore moiety (C_2_N_2_OS) is approximately planar, with a maximum deviation of −0.027 (1) Å, and is oriented at a dihedral angle of 86.7 (1)° with respect to the chloro­phenyl ring. An intra­molecular N—H⋯O hydrogen bond stabilizes the mol­ecular conformation. In the crystal, mol­ecules associate *via* N—H⋯S hydrogen bonds, forming inversion dimers with motif *R*_2_^2^(8). These dimers are further connected by N—H⋯O hydrogen bonds, forming *R*_2_^2^(12) dimers. As a result, hydrogen-bonded chains running along [110] are formed. C—H⋯S inter­actions also occur. The terminal two C atoms of the butyl chain are disordered over two positions with an occupancy ratio of 0.54:0.46.

## Related literature

For general background to the biological activity of thio­urea derivatives, see: Yang *et al.* (2012 ▶); Wu *et al.* (2012 ▶); Abbas *et al.* (2013 ▶); Ryu *et al.* (2012 ▶).
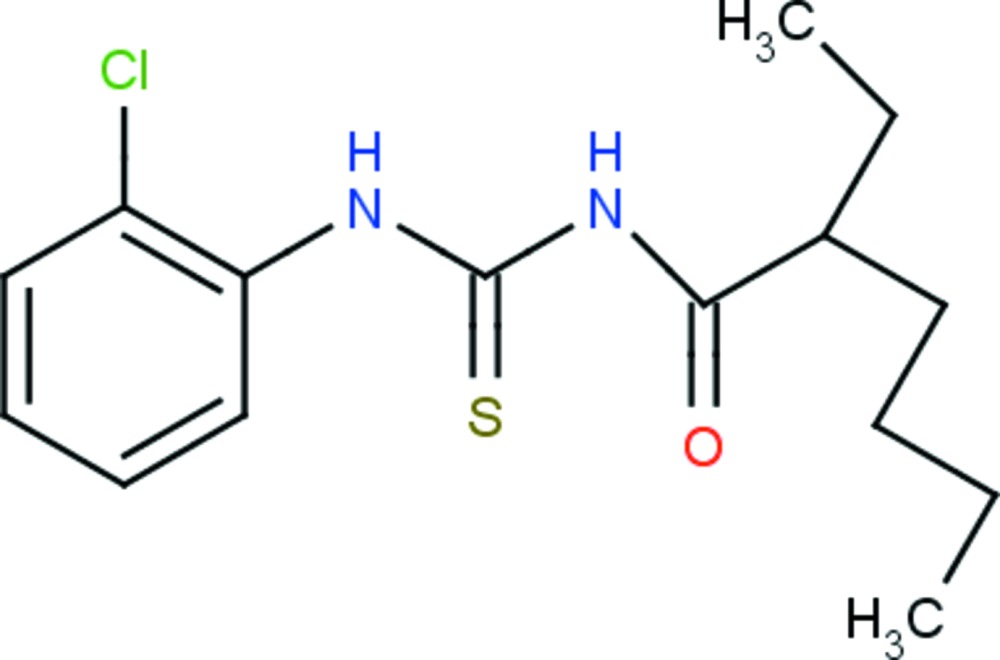


## Experimental

### 

#### Crystal data

C_15_H_21_ClN_2_OS
*M* *_r_* = 312.85
Triclinic, 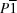

*a* = 7.264 (5) Å
*b* = 10.056 (7) Å
*c* = 11.935 (9) Å
α = 97.748 (17)°
β = 98.100 (17)°
γ = 103.72 (2)°
*V* = 825.5 (11) Å^3^
*Z* = 2
Mo *K*α radiation
μ = 0.36 mm^−1^
*T* = 292 K
0.22 × 0.20 × 0.18 mm

#### Data collection

Bruker SMART APEX CCD area-detector diffractometer
8136 measured reflections
2878 independent reflections
1700 reflections with *I* > 2σ(*I*)
*R*_int_ = 0.067

#### Refinement

*R*[*F* ^2^ > 2σ(*F* ^2^)] = 0.076
*wR*(*F* ^2^) = 0.230
*S* = 1.02
2878 reflections
200 parameters
4 restraints
H-atom parameters constrained
Δρ_max_ = 0.37 e Å^−3^
Δρ_min_ = −0.26 e Å^−3^

### 

Data collection: *SMART* (Bruker, 2001 ▶); cell refinement: *SAINT* (Bruker, 2001 ▶); data reduction: *SAINT*; program(s) used to solve structure: *SHELXS97* (Sheldrick, 2008 ▶); program(s) used to refine structure: *SHELXL2013* (Sheldrick, 2008 ▶); molecular graphics: *ORTEP-3 for Windows* (Farrugia, 2012 ▶) and *PLATON* (Spek, 2009 ▶); software used to prepare material for publication: *SHELXL97* and *PLATON*.

## Supplementary Material

Crystal structure: contains datablock(s) I, global. DOI: 10.1107/S160053681301828X/bt6917sup1.cif

Structure factors: contains datablock(s) I. DOI: 10.1107/S160053681301828X/bt6917Isup2.hkl

Supplementary material file. DOI: 10.1107/S160053681301828X/bt6917Isup3.cml

Additional supplementary materials:  crystallographic information; 3D view; checkCIF report

## Figures and Tables

**Table 1:** Hydrogen-bond geometry (Å, °)

*D*—H⋯*A*	*D*—H	H⋯*A*	*D*⋯*A*	*D*—H⋯*A*
N1—H1⋯O1	0.86	1.98	2.652 (4)	134
N1—H1⋯O1^i^	0.86	2.49	3.184 (5)	139
N2—H2⋯S1^ii^	0.86	2.61	3.451 (4)	168
C9—H9⋯S1^ii^	0.98	2.81	3.725 (5)	157

Symmetry codes: (i) 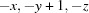; (ii) 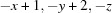.

**Fig. 1.**
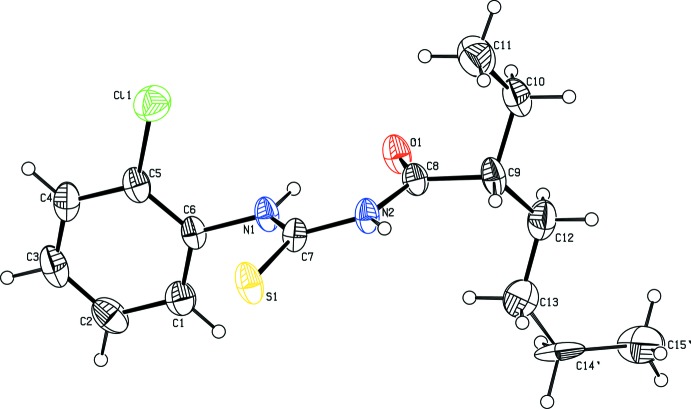
The molecular structure of the title compound, showing the atom-numbering scheme. Displacement ellipsoids are drawn at the 30% probability level. The minor occupied atoms of the disordered part have been omitted for clarity.

**Fig. 2.**
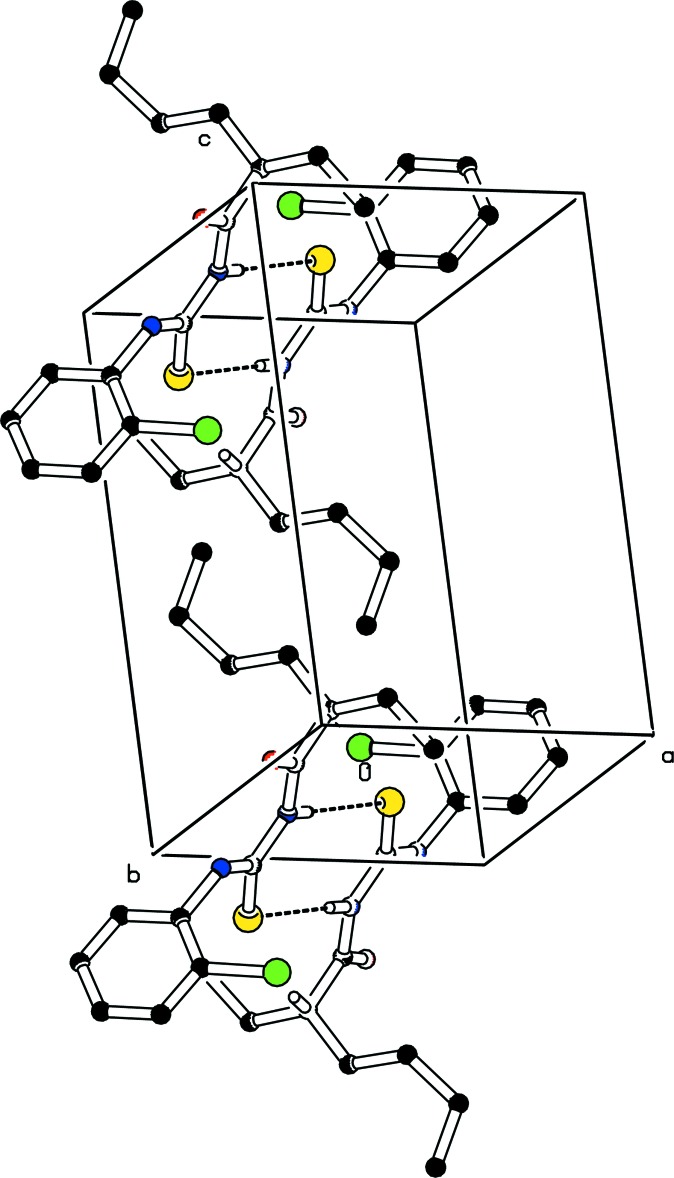
Molecular packing of the title compound, viewed along the b axis; H-bonds are shown as dashed lines. For the sake of clarity, H atoms, not involved in hydrogen bonds, have been omitted. The minor occupied atoms of the disordered part have been omitted for clarity.

**Fig. 3.**
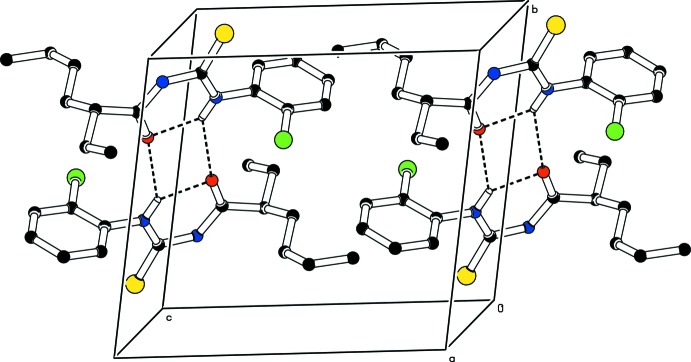
Molecular packing of the title compound, viewed down the a axis; H-bonds are shown as dashed lines. For the sake of clarity, H atoms, not involved in hydrogen bonds, have been omitted. Minor component of the disorder have been omitted for clarity.

## supplementary crystallographic information

### Comment

Thiourea derivatives are an important class of organic compounds in which sulfur is the major ligand atom which plays an important role in coordination chemistry. Thiourea derivatives possess a wide range of biological activities such as antibacterial (Yang *et al.*, 2012), antifungal (Wu *et al.*, 2012; Abbas *et al.*, 2013) activities. These derivatives sensitizes human H1299 lung carcinoma cells (Ryu *et al.*, 2012). In view of the biological importance of thioureas, we have undertaken a single-crystal X-ray diffraction study of the title compound, and the results are presented here.

The molecular structure and atomic connectivity for the title compound are illustrated in Fig. 1. The central chromophore moiety (C_2_N_2_OS) is planar with a maximum deviation of -0.027 (1) Å for atom C8. The dihedral angle between the chlorophenyl ring and the chromophor moiety is 86.7 (1)°.

The molecular structure is stabilized by an intramolecular N—H···O hydrogen bond (Table 1). In the molecular packing, N—H···S hydrogen bonds involving atoms N2 and S1 link inversion-related molecules to form R_2_^2^(8) graph set dimer (Fig. 2). These dimers are further connected by N—H···O hydrogen bonds forming R_2_^2^(12) dimers (Fig. 3). As a result of that, hydrogen bonded chains running along [110] are formed.

### Experimental

A mixture of supersaturated solutions of 2-chlorophenol (1 mmol), thiourea (1 mmol) and 2-ethylhexanoic acid (1mmol) were dissolved in ethanol (20ml). The mixture was stirred well and refluxed to 3hours. The reaction was ensured with a yellow crystalline solid deposited at the bottom of the beaker. Single crystals of (I) were obtained by slow evaporation method using ethanol as solvent at at room temperature.

### Refinement

H atoms were placed in idealized positions and allowed to ride on their parent atoms, with N—-H distance of 0.86 Å and C—H distances of 0.93-0.98 Å, and Uiso(H) = 1.5U_eq_(C) for methyl H atoms and Uiso(H) = 1.2U_eq_(C or N) for other H atoms. Atoms C14 and C15 are disordered over two positions with an occupancy of 0.46 and 0.54. The bond lengths of C13—C14 and C14—C15 are restrained to the value of 1.54 (1) Å.

### Crystal data

**Table:**

C_15_H_21_ClN_2_OS	*Z* = 2
*M**_r_* = 312.85	*F*(000) = 332
Triclinic, *P*1	*D*_x_ = 1.259 Mg m^−^^3^
*a* = 7.264 (5) Å	Mo *K*α radiation, λ = 0.71073 Å
*b* = 10.056 (7) Å	Cell parameters from 6222 reflections
*c* = 11.935 (9) Å	θ = 2.3–24.8°
α = 97.748 (17)°	µ = 0.36 mm^−^^1^
β = 98.100 (17)°	*T* = 292 K
γ = 103.72 (2)°	Block, colourless
*V* = 825.5 (11) Å^3^	0.22 × 0.20 × 0.18 mm

### Data collection

**Table:**

Bruker SMART APEX CCD area-detector diffractometer	*R*_int_ = 0.067
Radiation source: fine-focus sealed tube	θ_max_ = 25.0°, θ_min_ = 1.8°
ω scans	*h* = −8→8
8136 measured reflections	*k* = −11→11
2878 independent reflections	*l* = −14→14
1700 reflections with *I* > 2σ(*I*)	

### Refinement

**Table:**

Refinement on *F*^2^	Secondary atom site location: difference Fourier map
Least-squares matrix: full	Hydrogen site location: inferred from neighbouring sites
*R*[*F*^2^ > 2σ(*F*^2^)] = 0.076	H-atom parameters constrained
*wR*(*F*^2^) = 0.230	*w* = 1/[σ^2^(*F*_o_^2^) + (0.125*P*)^2^] where *P* = (*F*_o_^2^ + 2*F*_c_^2^)/3
*S* = 1.02	(Δ/σ)_max_ < 0.001
2878 reflections	Δρ_max_ = 0.37 e Å^−^^3^
200 parameters	Δρ_min_ = −0.26 e Å^−^^3^
4 restraints	

### Special details

**Table:**

Geometry. All esds (except the esd in the dihedral angle between two l.s. planes) are estimated using the full covariance matrix. The cell esds are taken into account individually in the estimation of esds in distances, angles and torsion angles; correlations between esds in cell parameters are only used when they are defined by crystal symmetry. An approximate (isotropic) treatment of cell esds is used for estimating esds involving l.s. planes.

### Fractional atomic coordinates and isotropic or equivalent isotropic displacement parameters (Å^2^)

**Table:**

	*x*	*y*	*z*	*U*_iso_*/*U*_eq_	Occ. (<1)
Cl1	0.0875 (2)	0.56892 (16)	−0.31845 (12)	0.0787 (5)	
S1	0.23733 (18)	0.95432 (13)	−0.12264 (10)	0.0649 (5)	
O1	0.1744 (4)	0.6032 (3)	0.0822 (3)	0.0589 (9)	
N1	0.0376 (5)	0.7093 (4)	−0.0934 (3)	0.0478 (9)	
H1	0.0249	0.6407	−0.0566	0.057*	
N2	0.3169 (5)	0.8104 (4)	0.0374 (3)	0.0480 (9)	
H2	0.4181	0.8790	0.0578	0.058*	
C1	−0.2560 (7)	0.7626 (5)	−0.1733 (4)	0.0581 (12)	
H1A	−0.2591	0.8089	−0.1010	0.070*	
C2	−0.4017 (7)	0.7532 (6)	−0.2651 (5)	0.0677 (15)	
H2A	−0.5034	0.7921	−0.2547	0.081*	
C3	−0.3938 (8)	0.6860 (6)	−0.3712 (5)	0.0733 (16)	
H3	−0.4911	0.6795	−0.4329	0.088*	
C4	−0.2470 (8)	0.6287 (5)	−0.3879 (4)	0.0645 (14)	
H4	−0.2441	0.5826	−0.4604	0.077*	
C5	−0.1014 (6)	0.6392 (5)	−0.2968 (4)	0.0514 (12)	
C6	−0.1079 (6)	0.7039 (4)	−0.1892 (3)	0.0425 (10)	
C7	0.1916 (6)	0.8164 (4)	−0.0593 (3)	0.0455 (11)	
C8	0.3017 (6)	0.7097 (5)	0.1054 (3)	0.0454 (10)	
C9	0.4490 (7)	0.7445 (5)	0.2141 (4)	0.0563 (13)	
H9	0.5358	0.8356	0.2147	0.068*	
C10	0.5689 (8)	0.6399 (6)	0.2138 (4)	0.0719 (15)	
H10A	0.6536	0.6587	0.2878	0.086*	
H10B	0.4831	0.5478	0.2056	0.086*	
C11	0.6883 (9)	0.6393 (7)	0.1219 (5)	0.094 (2)	
H11A	0.6062	0.6196	0.0480	0.142*	
H11B	0.7572	0.5693	0.1274	0.142*	
H11C	0.7783	0.7287	0.1312	0.142*	
C12	0.3511 (8)	0.7576 (6)	0.3165 (4)	0.0711 (15)	
H12A	0.2709	0.6665	0.3194	0.085*	
H12B	0.4495	0.7845	0.3853	0.085*	
C13	0.2284 (12)	0.8584 (8)	0.3208 (5)	0.110 (2)	
H13A	0.1449	0.8468	0.2473	0.132*	0.46
H13B	0.3081	0.9534	0.3400	0.132*	0.46
H13C	0.1402	0.8344	0.2478	0.132*	0.54
H13D	0.3138	0.9487	0.3212	0.132*	0.54
C14	0.104 (3)	0.8234 (19)	0.4181 (15)	0.109 (8)	0.46
H14A	−0.0231	0.8385	0.4033	0.131*	0.46
H14B	0.0991	0.7324	0.4382	0.131*	0.46
C15	0.251 (4)	0.941 (2)	0.499 (3)	0.187 (13)	0.46
H15A	0.2142	0.9492	0.5737	0.280*	0.46
H15B	0.2567	1.0259	0.4705	0.280*	0.46
H15C	0.3751	0.9218	0.5057	0.280*	0.46
C14'	0.105 (3)	0.882 (2)	0.4141 (13)	0.174 (14)	0.54
H14C	0.0756	0.9708	0.4128	0.209*	0.54
H14D	−0.0164	0.8103	0.3945	0.209*	0.54
C15'	0.197 (3)	0.879 (2)	0.5325 (13)	0.141 (10)	0.54
H15D	0.1141	0.8968	0.5853	0.211*	0.54
H15E	0.3176	0.9491	0.5529	0.211*	0.54
H15F	0.2200	0.7893	0.5360	0.211*	0.54

### Atomic displacement parameters (Å^2^)

**Table:**

	*U*^11^	*U*^22^	*U*^33^	*U*^12^	*U*^13^	*U*^23^
Cl1	0.0737 (10)	0.0888 (11)	0.0753 (9)	0.0267 (8)	0.0178 (7)	0.0058 (8)
S1	0.0619 (8)	0.0570 (8)	0.0629 (8)	−0.0092 (6)	−0.0105 (6)	0.0332 (6)
O1	0.056 (2)	0.054 (2)	0.0582 (19)	−0.0049 (17)	−0.0026 (15)	0.0277 (16)
N1	0.048 (2)	0.047 (2)	0.0413 (19)	−0.0009 (18)	−0.0026 (16)	0.0199 (17)
N2	0.046 (2)	0.047 (2)	0.0398 (19)	−0.0049 (16)	−0.0046 (15)	0.0137 (16)
C1	0.055 (3)	0.061 (3)	0.057 (3)	0.010 (2)	0.007 (2)	0.015 (2)
C2	0.045 (3)	0.066 (3)	0.093 (4)	0.012 (3)	0.004 (3)	0.029 (3)
C3	0.065 (4)	0.081 (4)	0.067 (4)	0.010 (3)	−0.014 (3)	0.030 (3)
C4	0.073 (4)	0.066 (3)	0.044 (3)	0.003 (3)	−0.005 (2)	0.014 (2)
C5	0.043 (3)	0.057 (3)	0.051 (3)	0.003 (2)	0.004 (2)	0.022 (2)
C6	0.035 (2)	0.045 (2)	0.042 (2)	−0.0019 (19)	−0.0005 (18)	0.016 (2)
C7	0.044 (2)	0.050 (3)	0.035 (2)	0.000 (2)	0.0018 (18)	0.010 (2)
C8	0.044 (2)	0.051 (3)	0.042 (2)	0.007 (2)	0.0069 (19)	0.018 (2)
C9	0.057 (3)	0.062 (3)	0.046 (3)	0.003 (2)	−0.003 (2)	0.028 (2)
C10	0.068 (4)	0.083 (4)	0.063 (3)	0.023 (3)	−0.008 (3)	0.023 (3)
C11	0.086 (4)	0.123 (6)	0.089 (4)	0.045 (4)	0.018 (4)	0.033 (4)
C12	0.090 (4)	0.069 (3)	0.046 (3)	0.006 (3)	0.004 (3)	0.014 (3)
C13	0.161 (7)	0.129 (6)	0.063 (4)	0.081 (6)	0.025 (4)	0.017 (4)
C14	0.113 (14)	0.079 (15)	0.15 (2)	0.065 (12)	0.018 (12)	−0.013 (12)
C15	0.14 (2)	0.12 (2)	0.27 (4)	0.027 (16)	−0.07 (2)	0.04 (2)
C14'	0.27 (3)	0.13 (2)	0.144 (18)	0.16 (2)	−0.006 (17)	−0.049 (15)
C15'	0.27 (3)	0.143 (17)	0.089 (11)	0.130 (19)	0.113 (15)	0.073 (12)

### Geometric parameters (Å, º)

**Table:**

Cl1—C5	1.720 (5)	C10—H10B	0.9700
S1—C7	1.655 (4)	C11—H11A	0.9600
O1—C8	1.206 (5)	C11—H11B	0.9600
N1—C7	1.326 (5)	C11—H11C	0.9600
N1—C6	1.430 (5)	C12—C13	1.501 (8)
N1—H1	0.8600	C12—H12A	0.9700
N2—C8	1.374 (5)	C12—H12B	0.9700
N2—C7	1.384 (5)	C13—C14'	1.553 (10)
N2—H2	0.8600	C13—C14	1.592 (10)
C1—C6	1.367 (6)	C13—H13A	0.9700
C1—C2	1.388 (7)	C13—H13B	0.9700
C1—H1A	0.9300	C13—H13C	0.9700
C2—C3	1.367 (7)	C13—H13D	0.9700
C2—H2A	0.9300	C14—C15	1.507 (10)
C3—C4	1.352 (7)	C14—H14A	0.9700
C3—H3	0.9300	C14—H14B	0.9700
C4—C5	1.379 (6)	C15—H15A	0.9600
C4—H4	0.9300	C15—H15B	0.9600
C5—C6	1.372 (6)	C15—H15C	0.9600
C8—C9	1.503 (6)	C14'—C15'	1.484 (10)
C9—C12	1.505 (7)	C14'—H14C	0.9700
C9—C10	1.516 (7)	C14'—H14D	0.9700
C9—H9	0.9800	C15'—H15D	0.9600
C10—C11	1.492 (8)	C15'—H15E	0.9600
C10—H10A	0.9700	C15'—H15F	0.9600
			
C7—N1—C6	122.2 (3)	C10—C11—H11C	109.5
C7—N1—H1	118.9	H11A—C11—H11C	109.5
C6—N1—H1	118.9	H11B—C11—H11C	109.5
C8—N2—C7	128.9 (4)	C13—C12—C9	116.9 (4)
C8—N2—H2	115.6	C13—C12—H12A	108.1
C7—N2—H2	115.6	C9—C12—H12A	108.1
C6—C1—C2	120.0 (5)	C13—C12—H12B	108.1
C6—C1—H1A	120.0	C9—C12—H12B	108.1
C2—C1—H1A	120.0	H12A—C12—H12B	107.3
C3—C2—C1	119.2 (5)	C12—C13—C14'	125.5 (8)
C3—C2—H2A	120.4	C12—C13—C14	105.6 (9)
C1—C2—H2A	120.4	C12—C13—H13A	110.6
C4—C3—C2	121.1 (5)	C14—C13—H13A	110.6
C4—C3—H3	119.4	C12—C13—H13B	110.6
C2—C3—H3	119.4	C14—C13—H13B	110.6
C3—C4—C5	119.6 (5)	H13A—C13—H13B	108.7
C3—C4—H4	120.2	C12—C13—H13C	105.9
C5—C4—H4	120.2	C14'—C13—H13C	105.9
C6—C5—C4	120.3 (5)	C12—C13—H13D	105.9
C6—C5—Cl1	120.0 (3)	C14'—C13—H13D	105.9
C4—C5—Cl1	119.7 (4)	H13C—C13—H13D	106.3
C1—C6—C5	119.7 (4)	C15—C14—C13	87.7 (19)
C1—C6—N1	119.8 (4)	C15—C14—H14A	114.0
C5—C6—N1	120.6 (4)	C13—C14—H14A	114.0
N1—C7—N2	116.1 (3)	C15—C14—H14B	114.0
N1—C7—S1	124.4 (3)	C13—C14—H14B	114.0
N2—C7—S1	119.5 (3)	H14A—C14—H14B	111.2
O1—C8—N2	122.6 (4)	C14—C15—H15A	109.5
O1—C8—C9	122.1 (4)	C14—C15—H15B	109.5
N2—C8—C9	115.3 (4)	H15A—C15—H15B	109.5
C8—C9—C12	109.7 (4)	C14—C15—H15C	109.5
C8—C9—C10	110.0 (4)	H15A—C15—H15C	109.5
C12—C9—C10	114.5 (4)	H15B—C15—H15C	109.5
C8—C9—H9	107.5	C15'—C14'—C13	114.4 (11)
C12—C9—H9	107.5	C15'—C14'—H14C	108.7
C10—C9—H9	107.5	C13—C14'—H14C	108.7
C11—C10—C9	115.2 (4)	C15'—C14'—H14D	108.7
C11—C10—H10A	108.5	C13—C14'—H14D	108.7
C9—C10—H10A	108.5	H14C—C14'—H14D	107.6
C11—C10—H10B	108.5	C14'—C15'—H15D	109.5
C9—C10—H10B	108.5	C14'—C15'—H15E	109.5
H10A—C10—H10B	107.5	H15D—C15'—H15E	109.5
C10—C11—H11A	109.5	C14'—C15'—H15F	109.5
C10—C11—H11B	109.5	H15D—C15'—H15F	109.5
H11A—C11—H11B	109.5	H15E—C15'—H15F	109.5
			
C6—C1—C2—C3	−0.7 (7)	C7—N2—C8—O1	5.4 (7)
C1—C2—C3—C4	0.0 (8)	C7—N2—C8—C9	−171.6 (4)
C2—C3—C4—C5	−0.6 (8)	O1—C8—C9—C12	−62.7 (6)
C3—C4—C5—C6	1.7 (7)	N2—C8—C9—C12	114.3 (4)
C3—C4—C5—Cl1	−178.9 (4)	O1—C8—C9—C10	64.0 (6)
C2—C1—C6—C5	1.8 (7)	N2—C8—C9—C10	−119.0 (4)
C2—C1—C6—N1	−177.5 (4)	C8—C9—C10—C11	64.0 (6)
C4—C5—C6—C1	−2.3 (7)	C12—C9—C10—C11	−172.0 (5)
Cl1—C5—C6—C1	178.3 (3)	C8—C9—C12—C13	−55.1 (6)
C4—C5—C6—N1	177.0 (4)	C10—C9—C12—C13	−179.3 (5)
Cl1—C5—C6—N1	−2.4 (6)	C9—C12—C13—C14'	176.0 (12)
C7—N1—C6—C1	−86.5 (5)	C9—C12—C13—C14	166.2 (9)
C7—N1—C6—C5	94.1 (5)	C12—C13—C14—C15	97.6 (12)
C6—N1—C7—N2	177.5 (4)	C14'—C13—C14—C15	−60 (3)
C6—N1—C7—S1	−1.0 (6)	C12—C13—C14'—C15'	39 (2)
C8—N2—C7—N1	−1.8 (7)	C14—C13—C14'—C15'	66 (4)
C8—N2—C7—S1	176.8 (4)		

### Hydrogen-bond geometry (Å, º)

**Table:**

*D*—H···*A*	*D*—H	H···*A*	*D*···*A*	*D*—H···*A*
N1—H1···O1	0.86	1.98	2.652 (4)	134
N1—H1···O1^i^	0.86	2.49	3.184 (5)	139
N2—H2···S1^ii^	0.86	2.61	3.451 (4)	168
C9—H9···S1^ii^	0.98	2.81	3.725 (5)	157

Symmetry codes: (i) −*x*, −*y*+1, −*z*; (ii) −*x*+1, −*y*+2, −*z*.

## References

[bb1] Abbas, S. Y., El-Sharief, M. A., Basyouni, W. M., Fakhr, I. M. & El-Gammal, E. W. (2013). *Eur. J. Med. Chem* **64**, 111–120. 10.1016/j.ejmech.2013.04.002 23644194

[bb2] Bruker (2001). *SMART* and *SAINT* Bruker AXS Inc., Madison, Wisconsin, USA.

[bb3] Farrugia, L. J. (2012). *J. Appl. Cryst.* **45**, 849–854.

[bb4] Ryu, B. J., Hwang, M. K., Park, M., Lee, K. & Kim, S. H. (2012). *Bioorg. Med. Chem. Lett.* **22**, 3862–3865. 10.1016/j.bmcl.2012.05.013 22622069

[bb5] Sheldrick, G. M. (2008). *Acta Cryst.* A**64**, 112–122. 10.1107/S0108767307043930 18156677

[bb6] Spek, A. L. (2009). *Acta Cryst.* D**65**, 148–155. 10.1107/S090744490804362X PMC2631630 19171970

[bb7] Wu, J., Shi, Q., Chen, Z., He, M., Jin, L. & Hu, D. (2012). *Molecules*, **17**, 5139–5150. 10.3390/molecules17055139 PMC6268560 22555301

[bb8] Yang, W., Liu, H., Li, M., Wang, F., Zhou, W. & Fan, J. (2012). *J. Inorg. Biochem.* **116**, 97–105. 10.1016/j.jinorgbio.2012.08.001 23018272

